# Monthly Report of Diseases

**Published:** 1800-10

**Authors:** 


### MONTHLY REPORT of DISEASES,

Admitted under the Care of the PHYSICIANS of the FINSBURY DISPENSARY, St. John's Square, Clerkenwell.
From Auguſt 20 to September 20, 1800.

| | | | |
|---|---|---|---|
| Continued Fever - - - | 54 | Chloroſis and Amenorrhœa | 10 |
| Small-Pox - - - - - - | 8 | Menorrhagia - - - - - | 3 |
| Cholera - - - - - - - | 20 | Cephalæa and Hemicrania | 11 |
| Diarrhœa and Dyſentery - | 62 | Dropſy - - - - - - - | 5 |
| Sore Throat - - - - - | 3 | Vertigo - - - - - - - | 2 |
| Rheumatiſm - - - - - - | 12 | Pleurodyne - - - - - - | 3 |
| Pleuriſy - - - - - - - | 1 | Nephralgia - - - - - - | 2 |
| Pulmonary Conſumption - | 5 | Colica Pictonum - - - - | 2 |
| Catarrh - - - - - - - | 1 | Hemiplegia - - - - - | 1 |
| Cough and Dyſpnœa - - | 8 | Eryſipelas - - - - - - | 1 |
| Aſthenia - - - - - - | 21 | Ptyaliſm - - - - - - - | 1 |
| Dyſpepſia - - - - - - | 6 | Urinæ Incontinentia - - | 1 |
| Gaſtrodynia and Enterodynia | 9 | Prurigo and Chronic Eruptions | 15 |
| Hypochondriaſis - - - | 3 | | |
| Hyſteria - - - - - - | 3 | Acute diſeaſes of Infants - | 18 |

Diſeaſes of the alimentary canal continue to be the reigning epidemics. The cholera, which was unuſually prevalent during the latter end of July, and the greater part of Auguſt, is now on the decline, and the diarrhœa and dyſentery have ſpread themſelves in an equal proportion; each diſorder thus obſerving, in the moft exact manner, its natural progrefs and feafon. The cholera rarely occurs in this country, except during the warmeft period of the fummer; and its frequency and violence correfpond, for the moft part, with the intenfity and duration of the atmofpherical heat. As a hot fummer immediately *excites* the cholera, fo it *predifpofes* to diarrhœa and dyfentery, which ufually make their appearance on the acceffion of the chilling damps of autumn. The immoderate ufe of fruit, to which thefe maladies are commonly attributed, may, in a few inftances, contribute to produce them; but in by far the greater number of cafes, there has been no reafon whatever, on minute enquiry, to afcribe their origin to matters taken into the ftomach. In conformity with the fame vulgar notion, it has been reported, that a quantity of damaged foreign wheat, faid to be mixed with the bread now made ufe of in London, has alfo had its fhare in promoting thefe diforders. The cholera of the prefent feafon, in feveral inftances, proved equally violent with that which is defcribed by Sydenham, as prevailing in the Summer of 1669; and it afforded fome idea of the feverity of this difeafe in the tropical regions. It often commenced with a very acute pain at the pit of the ftomach, or in fome part of the abdomen, which generally continued till the bilious vomiting and purging began to abate. Thefe evacuations, or ineffectual convulfive efforts to produce them, were almoft inceffant; and in the fhort intervals between them, the patients laid in the moft diftreffing ftate of naufea, pain, and anxiety, with an almoft total deprivation of mufcular power. The pulfe was much hurried, weak, and often irregular. The fkin was fcarcely hotter than natural, but frequently bedewed with a clammy moifture. The tongue was parched and foul, and there was a great complaint of thirft. In the majority of perfons, however, who were affected with this difeafe, it proved of a more mild nature; and in one only did it terminate fatally. The patient was a young woman, about 18 years of age, who for fome time before had been affected with a flight degree of leucophlegmatia, the confequence of amenorrhœa; after feveral hours of fevere vomiting and diarrhœa, fhe fuddenly became comatofe, and died in lefs than twenty-four hours afterwards. The violent ftraining in the act of vomiting had, probably, produced an effufion within the head, an accident rendered the more likely by the particular ftate of her habit.—The fymptoms of cholera afford a very good example of what phyficians call the vis medicatrix naturæ; and its medical treatment is founded on this principle: In general, it is only neceffary to give large quantities of mild liquids, in order to dilute the acrid bile, and to render its difcharge the more eafy. When the evacuations have ceafed, an opiate affords great comfort to the patient, by removing the naufea, pain, and uneafy feeling which remain, and by inducing a refrefhing fleep. When the difeafe, however, is extremely violent, or long continued, and is attended with great debility, it becomes expedient to add fome flight cordial to the liquid, which is drank, and to check the evacuations by means of opiates; fomentations to the ftomach and abdomen, may alfo be employed with advantage. A great irritability of the alimentary canal often remained for a confiderable time, the patient rejecting all kinds of aliment, having a foul tongue, and complaining of continual naufea. To remove thefe unpleafant confequences, an emetic was prefcribed, and for fome days afterwards aromatics, with fmall quantities of opium; and then the ufe of any light bitter foon reftored the ftomach to its ufual functions.—In others, a diarrhœa continued, and fometimes degenerated into a dyfentery. Indeed, the cholera, diarrhœa, and dyfentery, are very nearly allied, and pafs into each other by infenfible gradations; the two latter efpecially, are often fo much alike, that, except in a certain number of marked cafes, which point out the peculiar circumftances of their diftinction, it is doubtful whether we ought to affix to the difeafe the one name or the other. This, however, is of little importance in their treatment: In general, twelve or fifteen grains of rhubarb taken every morning for a few days, removes the complaint both fafely and effectually. A pill containing one grain of opium and one of ipecacuanha, may at the fame time be taken each evening on going to bed. The diet fhould confift of nourifhing emollient liquids, as weak broths, rice gruel, &c. In the cafes more purely dyfenteric, and attended with much pain and tenfion of the abdomen, a folution of neutral falts every morning has a more powerful effect than the rhubarb, in removing the fcybala retained in the inteftines. There were fome inftances of very fevere head-ach, and much general diforder occafioned by the diarrhœa having been prematurely checked by opiates and aftringents. In all cafes indeed of this complaint, but efpecially when it prevails epidemically, the means of ftopping it fhould be ufed with great caution.

The continued fever, or typhus, although confiderably increafed in frequency during the prefent month, has become more mild in its fymptoms; the furious delirium in particular, with which it was attended during the heat of the fummer, and which gave it at that time the name of the *brain fever*, having now, in good meafure, fubfided. A relaxed ftate of the bowels, at prefent, moft generally attends it; and, within due bounds, appears to be falutary. Thus, the obfervations of the immortal Sydenham, in regard to the influence of the reigning epidemic on the other contemporary difeafes, and the importance of keeping in view this fact in their medical treatment, receive, in every fucceeding feafon, additional confirmation.

